# Intelligent Microsystem for Sound Event Recognition in Edge Computing Using End-to-End Mesh Networking

**DOI:** 10.3390/s23073630

**Published:** 2023-03-31

**Authors:** Lulu Hou, Wenrui Duan, Guozhe Xuan, Shanpeng Xiao, Yuan Li, Yizheng Li, Jiahao Zhao

**Affiliations:** 1 School of Instrument Science and Opto-Electronics Engineering, Beijing Information Science and Technology University, Beijing 100192, China; 2 Department of Precision Instrument, Tsinghua University, Beijing 100084, China; 3 Key Laboratory of Smart Microsystem (Tsinghua University), Ministry of Education, Beijing 100084, China; 4 State Key Laboratory of Precision Measurement Technology and Instruments, Beijing 100084, China; 5 Beijing Laboratory of Biomedical Detection Technology and Instrument, Beijing 100084, China; 6 Beijing Advanced Innovation Center for Integrated Circuits, Beijing 100084, China; 7 China Mobile Research Institute, Beijing 100053, China; 8 The IoT Intelligent Microsystem Center, Tsinghua University-China Mobile Joint Research Institute, Beijing 100084, China

**Keywords:** wireless acoustic sensor network, intelligent microsystem, sound event recognition, edge computing, BLE mesh, acoustic signal processing, meshed network

## Abstract

Wireless acoustic sensor networks (WASNs) and intelligent microsystems are crucial components of the Internet of Things (IoT) ecosystem. In various IoT applications, small, lightweight, and low-power microsystems are essential to enable autonomous edge computing and networked cooperative work. This study presents an innovative intelligent microsystem with wireless networking capabilities, sound sensing, and sound event recognition. The microsystem is designed with optimized sensing, energy supply, processing, and transceiver modules to achieve small size and low power consumption. Additionally, a low-computational sound event recognition algorithm based on a Convolutional Neural Network has been designed and integrated into the microsystem. Multiple microsystems are connected using low-power Bluetooth Mesh wireless networking technology to form a meshed WASN, which is easily accessible, flexible to expand, and straightforward to manage with smartphones. The microsystem is 7.36 cm^3^ in size and weighs 8 g without housing. The microsystem can accurately recognize sound events in both trained and untrained data tests, achieving an average accuracy of over 92.50% for alarm sounds above 70 dB and water flow sounds above 55 dB. The microsystems can communicate wirelessly with a direct range of 5 m. It can be applied in the field of home IoT and border security.

## 1. Introduction

A Wireless Sensor Network (WSN) is a platform designed for efficient information acquisition that boasts quick deployment, wide coverage, and robust destruction resistance [1,2,3]. Sound signals are a rich source of information, and Wireless Acoustic Sensor Networks (WASNs) with sound event recognition capabilities are widely used in the field of Ambient Assisted Living (AAL), such as in smart homes [4]. Intelligent microsystems possess independent functionalities, including information acquisition, data processing, storage, communication, and energy harvesting [5]. Due to their advantageous features of intelligence, miniaturization, and affordability, intelligent microsystems are quintessential representatives of IoT terminals, garnering increasing attention [6,7].

Intelligent microsystems face severe limitations and interrelated constraints in practical applications and designs, including energy supply, communication, processing, sensing, and size. The utilization of high-performance processors and high-precision sensors increases the size and power consumption of microsystems. In contrast, the use of low-power devices, such as low-performing processors and low-precision sensors, impedes their intelligent sensing and processing capabilities [8]. Sending the data collected by microsystems to high-performance equipment for further processing results in an increase in communication costs and response time. Moreover, the sensing and communication range of a single microsystem is highly limited. As the number of IoT terminals grows, autonomous operation of microsystems and networking cooperation among microsystems become increasingly vital [9]. Thus, building a WASN with intelligent microsystems as nodes requires a balance between small size, low power consumption, sound sensing and recognition, and networked communication, making it a system optimization problem that warrants further research and practice.

Previous research has reported on some WASNs for sound event recognition [10]. However, a WASN proposed by [11] for noise measurement and sound event recognition in urban environments had poorly integrated sensing nodes and did not implement recognition locally. Other WASNs proposed for sound event recognition [12,13] utilized sensing nodes capable of local recognition assembled from microphones, Raspberry Pi, and power supplies. However, they had poor integration, large size, high power consumption, and high cost. Several reports have also focused on intelligent microsystems, including a 15 cm^3^ and 35 g microsystem for vibration sensing and target recognition [14]. Although the microsystem was well-integrated and could realize vibration wake-up and self-energy supply, its recognition accuracy was not optimal, and it did not implement wireless networking. Current research related to sound perception in intelligent microsystems has primarily focused on developing highly integrated, ultra-low-power sound sensor components, lacking effective system-level applications [15,16]. Zheng You presented an intelligent microsystem of 1.68 cm^3^ for sound source localization that consisted of a MEMS microphone and MCU, which had an excellent integration [17]. The acoustic source localization network consisted of four microsystem nodes with good localization accuracy, but the node access capability and scalability of the network had not been specified. To date, no intelligent microsystems with a small size, low power consumption, sound event recognition, and wireless networking capability have been reported.

**Figure 1:**
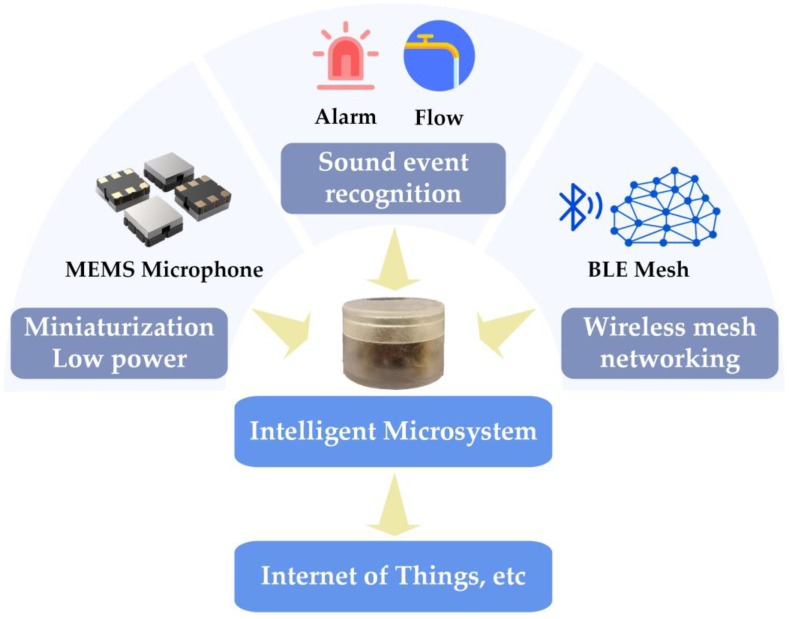
Overall working principle of the intelligent microsystem.

This paper presents the design and implementation of an intelligent microsystem capable of sound sensing, sound event recognition, and wireless networking. The hardware and algorithms were optimized to reduce size, weight, and power consumption while improving sound event recognition performance. Figure 1 illustrates the overall working principle of the microsystem, which integrates a MEMS microphone and MCU and is fully packaged. It is powered using a lithium battery and supports plug-and-play functionality. A CNN-based sound event recognition algorithm is designed and ported to the microsystem for edge computing, enabling high accuracy and responsive sound event recognition with MCU-level computing resources. Multiple microsystems form a mesh WASN using low-power Bluetooth (BLE) Mesh wireless networking technology, which can be managed by connecting to smartphones. The microsystem’s performance, including sound event recognition and networking communication, was verified with tests and experiments. This intelligent microsystem is suitable for various applications, such as smart homes, environmental monitoring, and unattended operations, as an effective IoT terminal. This is the first reported implementation of an intelligent microsystem with a small size, low power consumption, sound event recognition, and wireless networking capabilities.

## 2. Hardware Design

**Figure 2:**
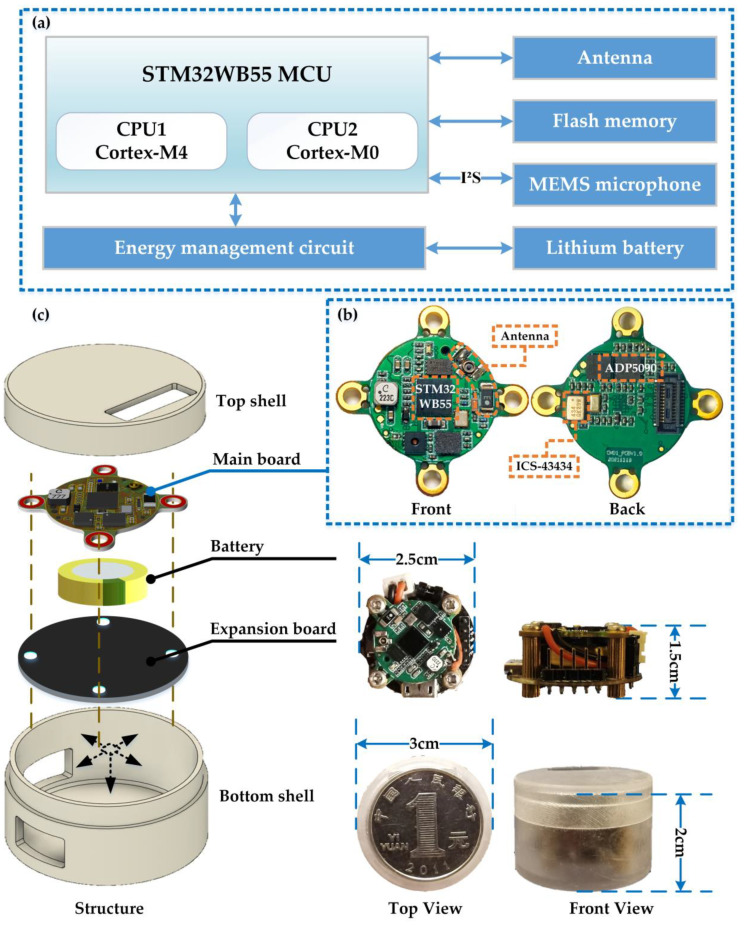
Hardware structure and components of the intelligent microsystem. (**a**) The composition of the microsystem. (**b**) The main board composition. (**c**) The microsystem hardware integration and packaging.

The hardware of the intelligent microsystem consists of four basic units: the sensing module, the processing module, the wireless transceiver module, and the power supply module. The composition of the microsystem is shown in Figure 2a. The selected main MCU is a low-power processor containing a Bluetooth chip for data processing and transmission. A MEMS microphone is used to capture sound signals and send sound data to the MCU using the I^2^S interface. An antenna for RF communication is integrated. The power supply module consists of a lithium battery and a supporting energy management circuit.

As shown in Figure 2b, the MCU, MEMS microphone, antenna, and energy management circuit are compactly integrated into a single PCB, called the main board, to reduce the size of the microsystem. To improve sound sensing quality and minimize noise interference, the microphone is positioned on the back of the main board while the sound hole on the front of the board faces the sound source. The MCU, a model STM32WB55, has two cores and supports multiple operating modes, including the sleeping mode and the active mode. The application processor CPU1 is an ARM Cortex-M4 core for overall control, and the network processor CPU2 is an ARM Cortex-M0 with the Bluetooth protocol stack. In addition, the MCU has a floating-point unit (FPU) and embedded memory (1 MB Flash, 256 KB SRAM) to meet the computing and processing requirements of this microsystem [14]. The ICS-43434 MEMS omnidirectional microphone has an impressive ultra-high 120 dB SPL AOP, as well as a high SNR of 64 dB, and a broadband frequency response of 60 Hz to 20 kHz. It supports high performance, low power, and sleep modes of operation and comes in a compact surface mount package measuring only 3.5 mm × 2.65 mm × 0.98 mm with a bottom sound hole. The MEMS microphone can directly communicate with the MCU using the I^2^S interface without an audio codec, allowing it to collect environmental sound and send the data to the MCU for processing based on the sampling rate set by the software program. The energy supply module uses a lithium battery for energy storage and power supply. The energy management circuit uses the ADP5090 chip to achieve energy management for charging and discharging, which also has over-discharge protection and over-charge protection to make the system safer and more reliable.

The microsystem hardware integration is shown in Figure 2c. The main board and battery are the core of the microsystem and can function independently. For experimental and debugging purposes, an extension board with plug-and-play and program debugging interfaces has been added. The main board and extension board are secured with four screws to form a skeleton, inside of which the lithium battery is placed. The housing is made of semi-transparent photosensitive resin material created using 3D printing, with holes for electrical connection and sound acquisition. Packaged as a cylinder, the microsystem is highly practical and capable of being powered either with wire for extended periods or with a lithium battery for portable applications. The size of the microsystem is presented in Table 1. In sleep mode, the microsystem consumes 0.18 mW, and this increases to 36 mW in active mode [14].

## 3. Software Design

### 3.1. Sound Event Recognition

**Figure 3:**
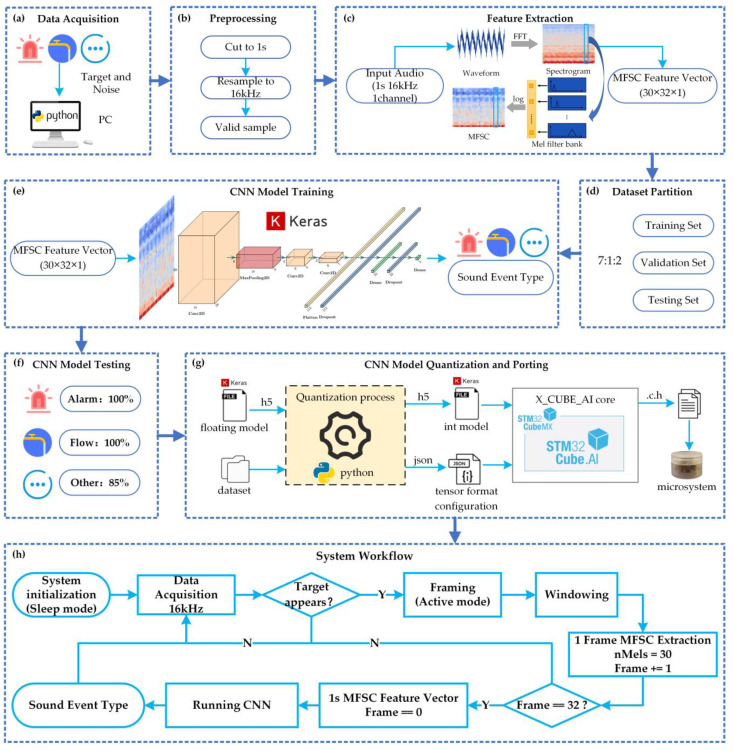
Sound event recognition algorithm and system workflow.

Alarm sounds indicate danger, and long-time water flow sounds represent a waste of water. In this paper, these two abnormal sound events are selected as recognition targets. To reduce interference and improve recognition accuracy, six types of sounds—door knocking, mouse-clicking, keyboard tapping, door creaking, washing machine running, and indoor background sound—are added to the recognition model as noise. Due to the limited sensing and computing resources of the intelligent microsystem, a simple and efficient sound event recognition algorithm and system workflow are designed to achieve accurate and responsive recognition of sound events, as shown in Figure 3.

#### 3.1.1. Data Acquisition and Preprocessing

Figure 3a depicts the collection of data from two sources. The first was a public dataset named ESC-50 [18], which comprises 2000 environmental recordings of 50 types of sounds, each with a duration of 5 s, a sampling rate of 44.1 kHz, and a single channel. The sounds of interest, including alarm, door knocking, mouse-clicking, keyboard tapping, door creaking, and washing machine running, were extracted from ESC-50. The other source was an indoor experimental dataset, containing recordings of water flow sounds and indoor background sounds, captured with smartphones. The recordings in this dataset vary in duration, have a sampling rate of 48 kHz, and consist of two channels.

In order to ensure sample consistency, responsive recognition, and compliance with the microsystem processing capability, the data were pre-processed, as shown in Figure 3b. This involved segmenting the data into new samples with a duration of 1 s, a single channel, and resampling it to a rate of 16 kHz. It is worth noting that some of the new samples may not contain valid sounds and will need to be filtered out.

To ensure balance in the different types of sound samples, the authors used 300 samples for each of the water flow and alarm sounds as target samples, and 50 samples for each of the other six types of sounds, totaling 300 samples, as noise samples. Both target and noise samples were used for model training and testing. As there were fewer valid samples of water flow and alarm sounds, data augmentation was necessary, which not only expanded the dataset but also helped the classification model avoid overfitting [19,20]. For this study, data augmentation was performed by altering the timing and pitch of the sound samples.

#### 3.1.2. Feature Extraction

The formula for converting ordinary frequency to mel-frequency is mel(f) = 2595 × log10(1 + f/700), where f is the frequency of the sound signal in Hertz (Hz).

The flow of MFSC feature extraction is shown in Figure 3c. First, the sound signal is framed and windowed. Then, the spectrum is obtained using fast Fourier transform (FFT) for each short-time window. Next, the Mel filter bank is used to convert the sound from the spectral domain to the Mel spectral domain. For non-vocal sounds, a Mel filter bank with the same bank height is selected [21], and the number of Mel filters is set to 30. Finally, the MFSC feature is obtained using natural logarithm processing of the Mel spectrum.

Using this process, sound data of 1 s duration and 16 kHz sampling rate can be transformed into a (30,32,1) MFSC feature vector, significantly reducing the number of operations and complexity of the recognition model input.

#### 3.1.3. CNN Model Training

Artificial neural networks are commonly used for pattern recognition, and one of the most well-known networks is the Convolutional Neural Network (CNN) [22]. Compared to traditional machine learning algorithms, a CNN has good self-learning, fitting, and generalization abilities, as well as a simple structure and few parameters, making it easier to optimize the model. Additionally, a CNN has parallel processing and fast computing capabilities, making it suitable for edge computing devices [23,24]. Therefore, in this paper, a CNN is chosen as the classification model for sound event recognition, and the model is designed, debugged, and evaluated using the artificial neural network library Keras.

Figure 3d shows that the dataset is divided into training, validation, and test sets with a ratio of 7:1:2. The model training process is illustrated in Figure 3e. The model takes the MFSC feature vector as input and outputs the probability of each sound event type. To suit the model’s intended use in the microsystem, a CNN with a simple structure is designed with parameters as indicated in Table 2. The processing complexity of the model is represented by the MACC [25], which is a unit of multi-ply-and-accumulate complexity.

**Figure 4:**
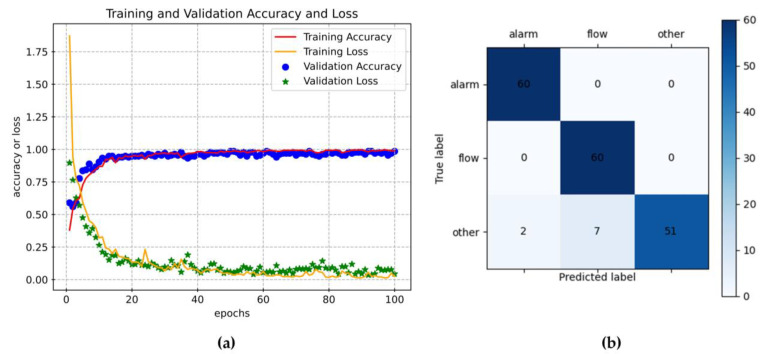
CNN model training and testing. (**a**) Model training and validation accuracy and loss. (**b**) The confusion matrix in model testing.

The model training process is illustrated in Figure 4a. After 100 epochs of training, the accuracy of both the training set and the validation set approaches 99%. Figure 4b shows the confusion matrix for the model test. The average accuracy of the test set is 95%, with the alarm sound and water flow sound achieving 100% accuracy, while the accuracy for other sounds is 85%.

#### 3.1.4. Model Quantification and Porting

Due to limited computational resources in the microsystem, deploying the trained 32-bit floating-point model with large memory usage directly into the microsystem is not feasible. Therefore, the model needs to be converted into an 8-bit fixed-point model using quantization. Quantization involves establishing a mapping relationship between floating-point and fixed-point data with post-training quantization (PTQ) and quantization awareness training (QAT) being the commonly used quantization methods [26]. PTQ is relatively simple and allows the quantization of a pre-trained model with a limited representative dataset. On the other hand, QAT is more complex but yields better model accuracy by performing quantization during the training process.

As illustrated in Figure 3g, this paper performs 8-bit PTQ and ports the trained CNN model based on STM32 X_CUBE_AI. As shown in Table 3, the quantized model has a 0.25% decrease in accuracy, a 7.99% decrease in complexity, a 71.94% decrease in peak RAM usage, and a 73.98% decrease in peak flash usage. By trading off a small loss in accuracy, quantization achieves the goal of compressing model parameters, reducing model memory usage, lowering model complexity, and improving model running speed. Finally, the quantized model is converted into C files containing the model structure, weight parameters, and calling interfaces, which are then deployed to the microsystem to recognize sound events.

#### 3.1.5. System Workflow

To optimize the software workflow of the microsystem, given the limited hardware resources and sound event recognition performance, several steps have been taken. These include system initialization, event initial detection, system work mode switching, preprocessing, feature extraction, and event recognition, as depicted in Figure 3h.

During system initialization, the microsystem determines the signal sampling period, microphone sampling rate, and ambient noise threshold before entering the low-power sleep mode. In this mode, the microphone remains active for sound detection, while the rest of the microsystem operates at a low-power state.

Event initial detection relies on sound amplitude to determine the presence of an event. If the sum of sound amplitudes in a sampling period is greater than five times the ambient noise threshold or twice the sum value of the previous sampling period, the microsystem switches to the high-power active mode. During this detection process, sound signals are continuously collected at a sampling rate of 16 kHz. A frame is made up of 1024 sampling points, with a frameshift of 512 sampling points. The Hanning window is used, and the window length is the same as the frame length.

Mel filtering of one frame of data outputs 30 Mel spectral coefficients, and a 1 s sound contains 32 frames. Therefore, the microsystem performs 32 cycles of processing to obtain the MFSC feature vector of a 1 s sound. This feature vector is used as input for a CNN classifier, which is run to obtain the sound event type.

### 3.2. Wireless Mesh Networking

The microsystem can recognize sound events independently, but its communication and sensing range is limited, so it needs to be networked with other microsystems. Topologies of WSNs include the star, tree, mesh, hierarchical topologies, etc. Meshed networks are also known as “multi-hop” networks, in which nodes communicate in a many-to-many manner, with the advantages of redundancy and reliability [27,28]. The common mesh networking technologies include BLE mesh [29], WIFI mesh [13], and ZigBee mesh [27]. BLE mesh has the advantages of self-organization, high security, simple routing, low power consumption, and a high device support [29]. Therefore, in this paper, BLE mesh is used as a networking method for the microsystems.

#### 3.2.1. BLE Mesh Theory

**Figure 5:**
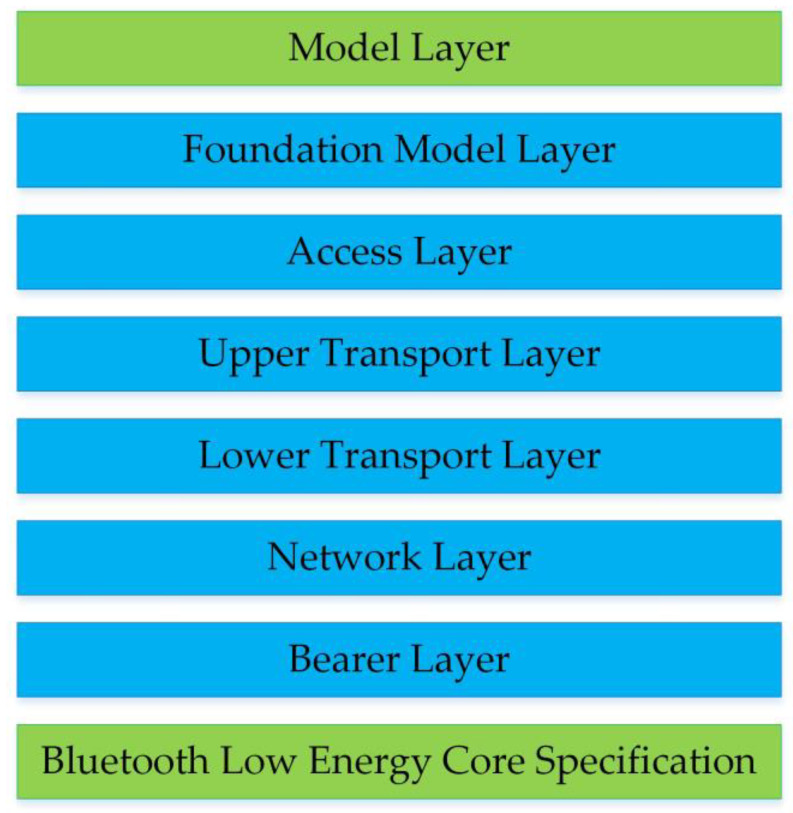
The BLE mesh architecture.

BLE mesh is a Bluetooth Low Energy-based networking specification published by Bluetooth SIG [29]. It uses the “flooding” method [30] and the publish/subscribe mechanism [31] to transmit messages. There are different types of nodes: relay, proxy, friend, and low-power. Relay nodes retransmit received messages, and they enable multiple “hops” in the network. Proxy nodes expose the interface for a smartphone or other devices to interact with the mesh network. A BLE mesh network has a theoretical maximum of 32,767 nodes and up to 126 hops [31]. BLE mesh defines four address types, three of which are used for message delivery: unicast addresses, virtual addresses, and group addresses [32]. The architecture of BLE mesh is shown in Figure 5, where the model layer defines the functions and behaviors of the nodes [31]. From the control perspective, the models can be divided into Server, Client, and Control models. Server models expose their state to other models, Client models can access the state of Server models, and Control models are a combination of them. From the functional perspective, there are Generic models, Lighting models, Sensor models, and Vendor models.

#### 3.2.2. Design and Implementation

**Figure 6:**
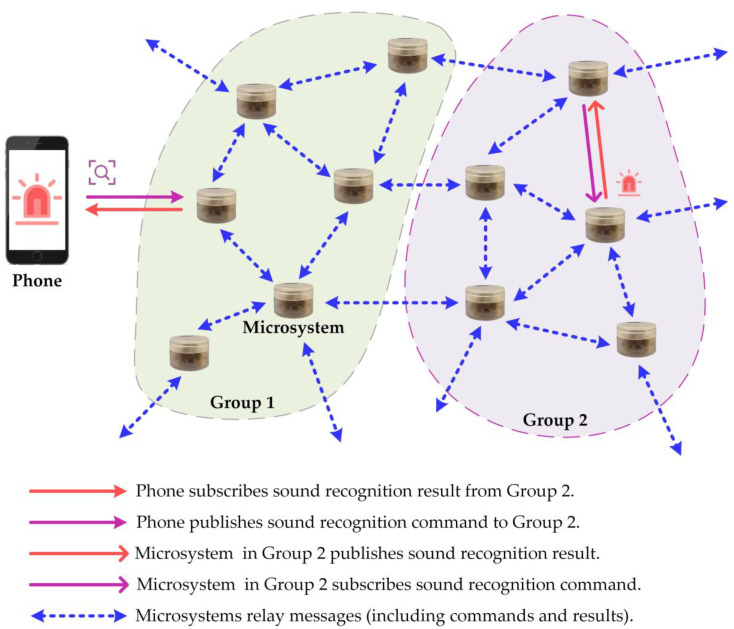
Design and implementation of the end-to-end mesh networking.

The STM32WB55 MCU has been selected for its ability to support BLE mesh technology. Figure 6 illustrates how multiple microsystems can be networked together, enabling control and monitoring of sound event recognition results using a mobile device. The network is easily accessible, flexible to expand, and straightforward to manage. Each microsystem includes relay and proxy features, as well as Sensor Server models for sensing and Vendor models for control.

When connected to the network, the microsystem nearest to the smartphone within the direct Bluetooth communication range acts as a proxy node. The smartphone can publish messages, such as recognition commands, which the microsystems can receive if subscribed. The microsystems receive the recognition command and perform sound event recognition locally, then publish the sound event recognition results. Different microsystems can subscribe to specific message groups, such as those organized by floor. Messages can be relayed between any two microsystems within the direct Bluetooth communication range, allowing for flexible management of microsystems within the network and enabling monitoring of sound events in any location within the network coverage.

As depicted in Figure 6, a mesh network is formed by several microsystems and mobile phones, which are divided into two groups. In this configuration, Group 2 receives sound event recognition instructions from the phone and subscribes to the sound event recognition results. The sound recognition command is transmitted to the proxy microsystem that is directly connected to the phone. The “command” message is then relayed to the microsystem of Group 2 using several microsystems. The microsystem of Group 2 subscribes to the recognition command, performs sound event recognition locally, and publishes the sound event recognition results. The “recognition results” message is then relayed to the phone via several microsystem relays, enabling sound event monitoring.

## 4. Results and Discussion

In the sound event recognition experiment, the performance of microsystem sound event recognition in edge computing was tested using alarm and water flow sounds as targets.

In the networking communication experiment, the communication range between a smartphone and a microsystem, as well as the relay range between two microsystems, were tested.

The networking recognition experiment involved deploying multiple microsystems indoors to verify their networking communication and generalization recognition capabilities.

### 4.1. Sound Event Recognition Experiment

**Figure 7:**
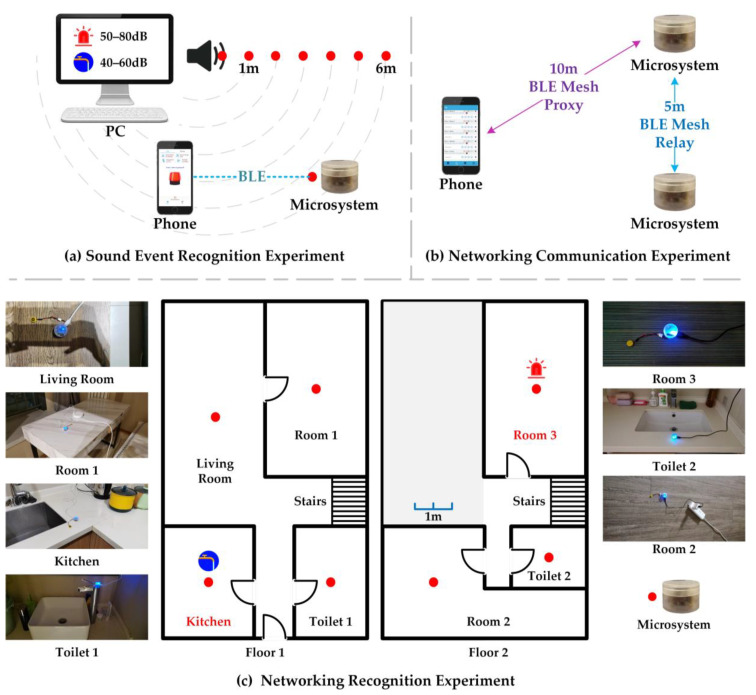
Overall experimental design.

In the sound event recognition experiment, 25 alarm audios and 25 water flow audios from the dataset described in Section 3.1.1, each 5 s long, were used as experimental samples. The microsystem’s recognition accuracy was tested indoors at different distances from the sound source and with different sound intensities, as shown in Figure 7a.

**Figure 8:**
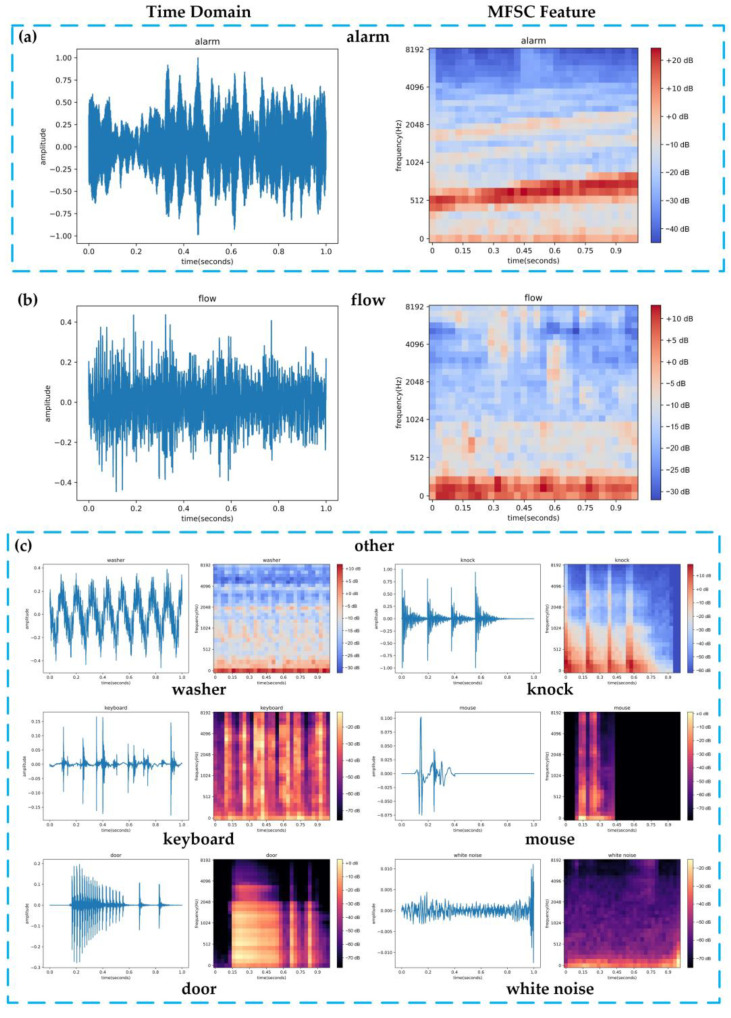
Distribution of sound signals in the time domain and MFSC features.

Three sound samples, namely an alarm sound (shown in Figure 8a), a water flow sound (shown in Figure 8b), and noise samples (shown in Figure 8c), were randomly selected from the experimental dataset. The time domain distribution and MFSC features of these sound signals are presented in Figure 8.

While the amplitude of the alarm sound is greater than that of the water flow sound, this feature alone cannot distinguish between the two types of sound events since the distance between the microsystem and the sound source is flexible. However, the MFSC features of the alarm sound and water flow sound show clear differentiation. The frequency of the alarm sound is mainly concentrated above 500 Hz, while the frequency of the water flow sound is mostly concentrated between 0 and 100 Hz.

**Figure 9:**
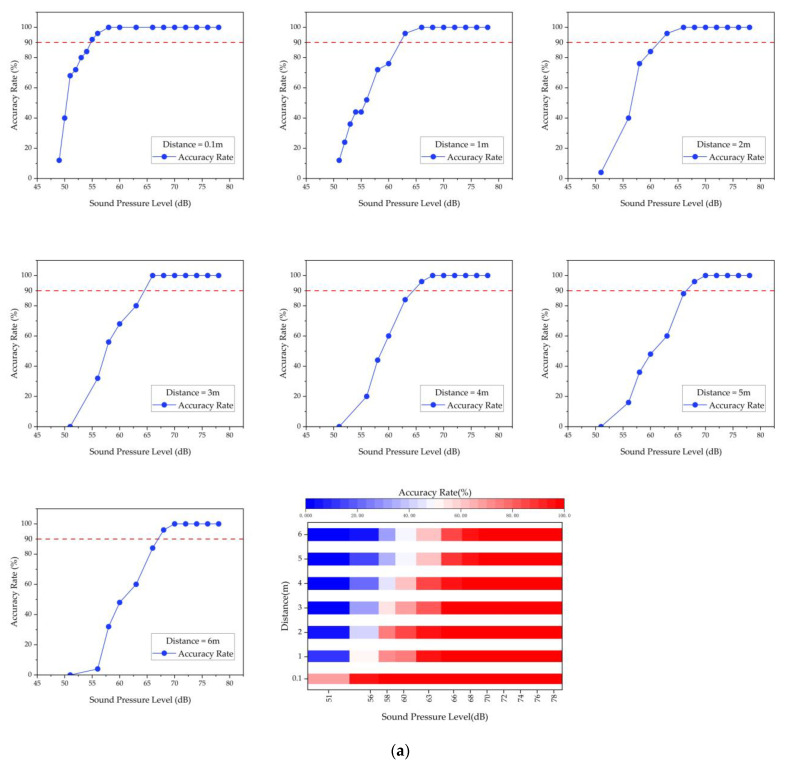

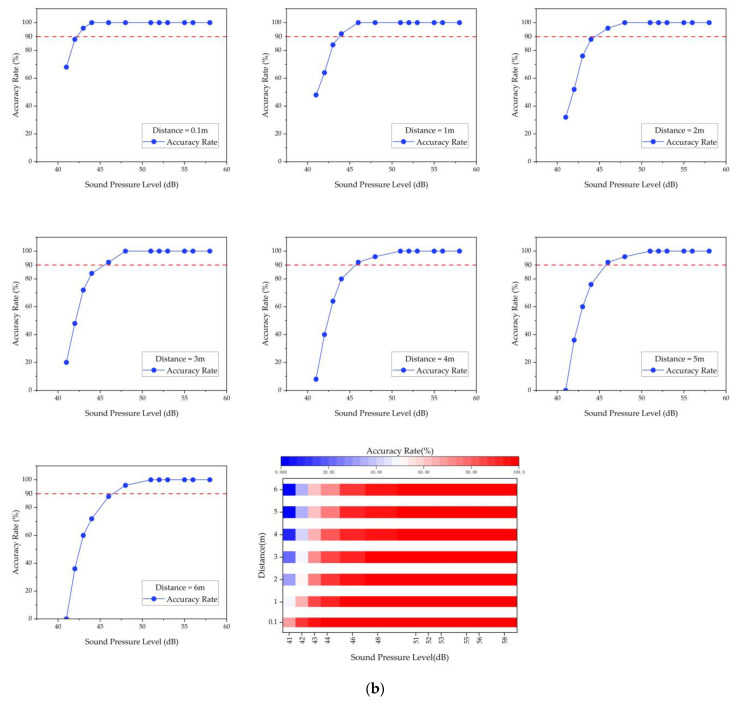
(**a**) Recognition results for the alarm sound. (**b**) Recognition results for the water flow sound. The red dashed line in the figure represents a recognition accuracy of 90%.

Figure 9 shows that the microsystem’s recognition accuracy improves as the distance between the microsystem and the sound source decreases and as the intensity of the sound source increases. As shown in Table 4, when tested indoors with low noise interference using the trained data, the microsystem can effectively identify sound events within 6 m with 100% accuracy and an average recognition time of no more than 3 s when the alarm sound is greater than 70 dB and the water flow sound is greater than 50 dB.

This suggests that the microsystems are capable of responsive and accurate sound event recognition. It also confirms the detection range of a single microsystem, which is important information for the networking communication experiment.

### 4.2. Networking Communication Experiment

In the networking communication experiment, the aim was to network the smartphone and the microsystems. The smartphone would send control commands to the microsystems while the microsystems would send sound event recognition results to the smartphone. As shown in Figure 7b, the range of direct communication between the smartphone and a single microsystem is around 10 m, while the direct communication distance between two microsystems is around 5 m.

### 4.3. Networking Recognition Experiment

Generalizability refers to a model’s ability to accurately predict on new, previously unseen data after being trained on a particular dataset. To evaluate the effectiveness and generalization of the sensing network in practical applications, a new test dataset is constructed, comprising audio samples of alarm sounds and water flow that were not used in model training. The sample parameters for the test dataset are shown in Table 5.

Seven microsystems were deployed in a house according to confirmed relay distances, as shown in Figure 7c, which illustrates the layout of the house and the location of the microsystems. Tests were conducted to recognize water flow sounds in Floor1-Kitchen and alarm sounds on Floor2-Room3, and the smartphone communication to the sensing network was verified once again. The sound source distance for this experiment was set to 0.1 m, 1 m, 2 m, and 3 m, considering that the communication distance between the two microsystems is approximately 5 m.

**Figure 10:**
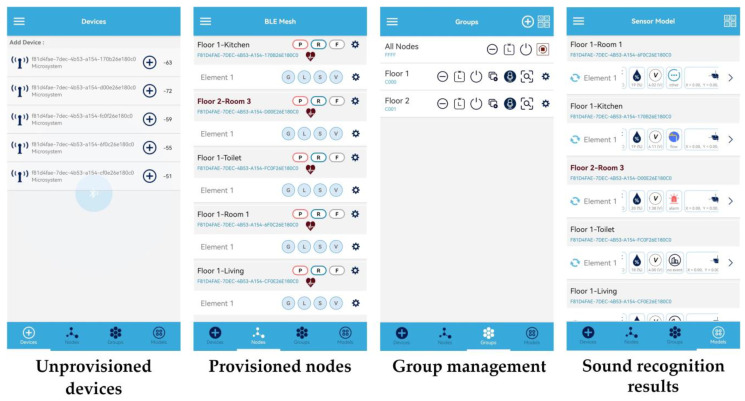
The interaction process between the smartphone and the microsystem sensing network.

Figure 10 illustrates the interaction process between the smartphone and the microsystem sensing network, which proceeds as follows: scanning the unprovisioned microsystems, configuring them as network nodes, controlling them by group, and receiving the sound event recognition results from the microsystems. From anywhere in the house, the smartphone could monitor the sound event recognition results of all microsystem nodes in real time. The smartphone could also control this meshed network in a flexible manner, such as controlling a microsystem individually, controlling multiple microsystems in groups (e.g., by floor), or controlling all microsystems in the house. This demonstrates that the end-to-end meshed network based on BLE mesh and the microsystems has good topological flexibility and network scalability while also effectively extending the detection range.

**Figure 11:**
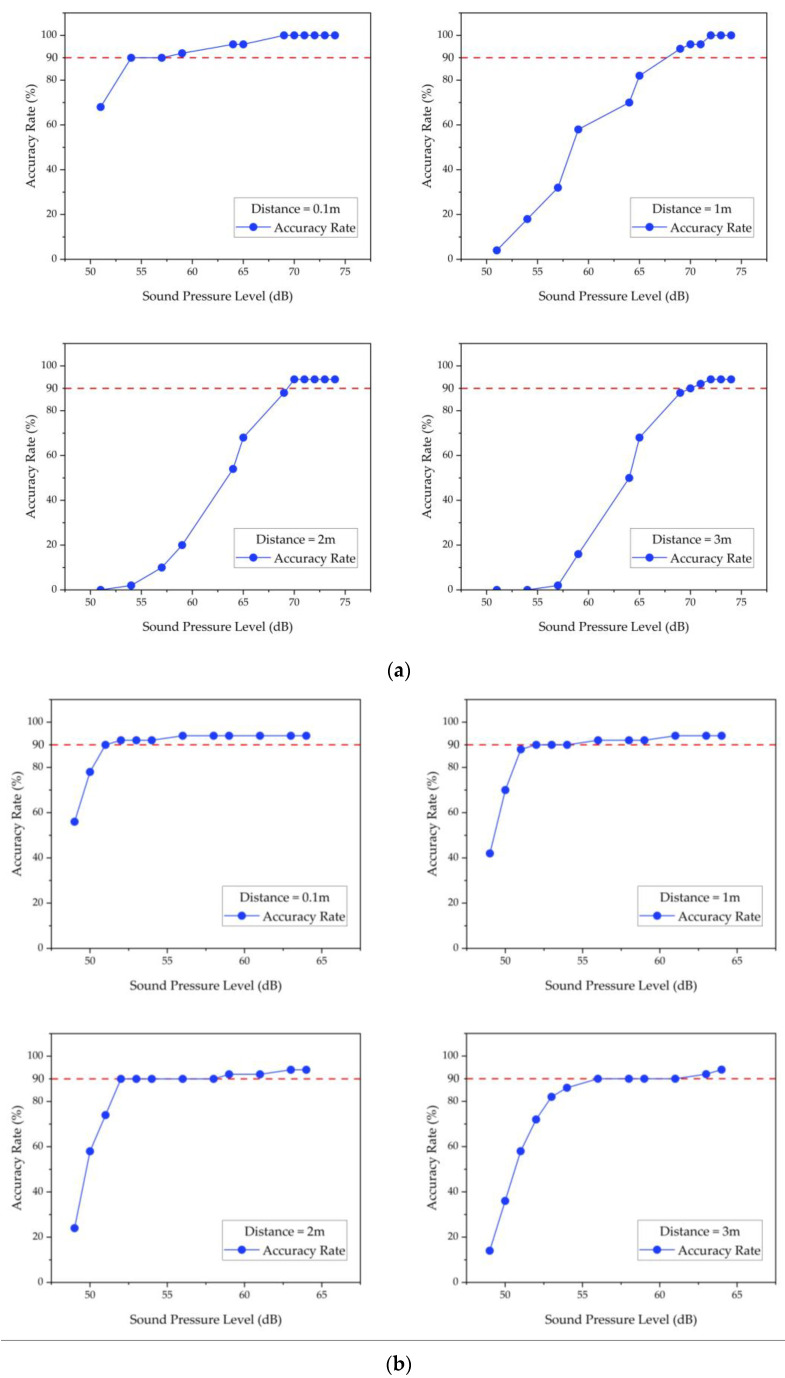
(**a**) Alarm sound recognition results in Floor2-Room3. (**b**) Water flow sound recognition results in Floor1-Kitchen. The red dashed line in the figure represents a recognition accuracy of 90%.

The effective recognition standard was set to achieve a recognition accuracy rate greater than 90% within a range of 3 m. As illustrated in Figure 11, the sound intensity of the target sound event increases as the distance between the microsystem and the sound source decreases, resulting in higher recognition accuracy. According to Table 6, sound events, such as alarm sounds with a sound intensity greater than 70 dB, and water flow sounds with a sound intensity greater than 55 dB can be effectively recognized with an average recognition accuracy rate of 96.30% and 92.50%, respectively. This demonstrates that the proposed sound event recognition approach is effective and has general applicability.

## 5. Conclusions

This paper presents the design and implementation of an intelligent microsystem with sound perception, sound event recognition, and wireless networking capabilities. The hardware modules are optimized for small size, lightweight, and low power consumption. The microsystem, which can be powered with a lithium battery and supports plug-and-play, has a volume of 7.36 cm^3^ and a weight of 8 g without housing and 14.14 cm^3^ and a weight of 13 g with housing. The software includes a low-computation sound event recognition algorithm based on a CNN and an end-to-end wireless mesh network based on BLE mesh.

In sound event recognition testing, the microsystem demonstrated a high level of accuracy and responsiveness. When tested with trained samples and the sound source within 6 m, the microsystem achieved an average recognition accuracy of 100% for alarm sounds greater than 70 dB and water flow sounds greater than 50 dB, with an average recognition time of no more than 3 s. When tested with untrained samples within 3 m of the sound source, the microsystem achieved an average recognition accuracy of 96.30% for alarm sounds greater than 70 dB and 92.50% for water flow sounds greater than 55 dB. In wireless mesh networking, the direct communication range between microsystems is 5 m, and this meshed network can be monitored and controlled with devices such as smartphones.

This intelligent microsystem and its wireless meshed network have made progress in responsive and accurate recognition of sound events, size reduction, power consumption reduction, topology flexibility increase, and device adaptability improvement. This work can be used as the sensing layer of IoT and applied to various intelligent environment applications, such as smart homes and unattended surveillance networks. The presented intelligent microsystem is a promising solution for sound event recognition and wireless networking in intelligent environments.

## Figures and Tables

**Table 1:** Microsystem size.

Size	Core	Without Housing	With Housing
Volume (cm^3^)	0.88	7.36	14.14
Weight (g)	4.00	8.00	13.00

**Table 2:** The CNN model structure.

ID	Layer Type	Output Shape	Complexity (MACC)
1	Input	(30,32,1)	N/A
2	Conv2D	(28,30,16)	120,976
	Nonlinearity	(28,30,16)	13,440
3	BatchNormalization	(28,30,16)	N/A
4	MaxPooling2d	(9,6,16)	12,960
5	Conv2D	(9,4,8)	13,832
	Nonlinearity	(9,4,8)	288
6	Conv2D	(7,2,8)	8072
	Nonlinearity	(7,2,8)	112
7	BatchNormalization	(7,2,8)	N/A
8	Flatten	(1,1,112)	N/A
9	Dropout	(1,1,112)	N/A
10	Dense	(1,1,32)	3616
	Nonlinearity	(1,1,32)	32
11	Dropout	(1,1,32)	N/A
12	Dense	(1,1,3)	99
	Nonlinearity	(1,1,3)	45

N/A refers to the layer whose complexity is negligible.

**Table 3:** Comparison between the original model and the quantified model.

Model Type	Accuracy (%)	Complexity (MACC)	Ram Usage (KB)	Flash Usage (KB)
Original model	95.24	173,472	12.76	18.95
Quantified model	94.99	159,606	3.58	4.93

**Table 4:** Results of the sound event recognition experiment.

Sound Event Type	Average Recognition Accuracy (%)							
	0.1 m	1 m	2 m	3 m	4 m	5 m	6 m	Total
Alarm (51~78 dB)	97.00	84.00	83.33	78.00	75.33	70.33	68.67	79.52
Alarm (70~78 dB)	100.00	100.00	100.00	100.00	100.00	100.00	100.00	100.00
Flow (41~58 dB)	96.00	90.67	87.00	84.67	81.67	80.00	79.33	85.62
Flow (50~58 dB)	100.00	100.00	100.00	100.00	100.00	100.00	100.00	100.00

**Table 5:** Sample parameters of the networking recognition experiment.

Sound Event Type	Sample Size	Sample Duration (s)	Average Sound Pressure Level (dB)	Sampling Rate (kHz)	Channels
Alarm	50	5	51~74	44.1	2
Flow	50	5	49~64	48	2

**Table 6:** Results of the networking recognition experiment.

Sound Event Type	Average Recognition Accuracy (%)				
	0.1 m	1 m	2 m	3 m	Total
Alarm (51~74 dB)	94.33	70.83	59.33	57.33	70.46
Alarm (70~74 dB)	100.00	98.40	94.00	92.80	96.30
Flow (49~64 dB)	88.67	85.67	81.50	74.50	82.59
Flow (56~64 dB)	94.00	93.00	92.00	91.00	92.50

## Data Availability

The data presented in this study are openly available at [https://doi.org/10.1145/2733373.2806390], reference number [18]. This data can be found here: [https://github.com/karolpiczak/ESC-50].
